# Real-Time Influenza Surveillance Using DeepSeek-Augmented BERT Classification on Chinese Social Media: Infodemiology Study

**DOI:** 10.2196/98164

**Published:** 2026-10-27

**Authors:** Shikai Chen, Chai Zhang, Xiyi Chen, Yan Zhao, Yuanyuan Zhang, Hao Zhang, Xiaofei Bian

**Affiliations:** 1 School of Public Health, Dalian Medical University, 9 West Section, Lvshun South Road, Lvshunkou District, Dalian, Liaoning, 116044, China, 86 18369448007; 2 The Second Hospital of Dalian Medical University, Dalian, Liaoning, China; 3 Dalian Rehabilitation and Recuperation Center of Joint Logistics Support ForceDalian, Liaoning, China; 4 Department of Pediatrics, Dalian Medical University, Dalian, Liaoning, China

**Keywords:** influenza surveillance, social media mining, large language models, bidirectional encoder representations from transformers, BERT, digital epidemiology, nowcasting

## Abstract

**Background:**

Traditional influenza surveillance systems rely on sentinel hospitals and often experience a 1‐ to 2-week reporting lag, limiting timely public health response. Social media platforms provide real-time data streams, but extracting reliable epidemiological signals remains challenging due to substantial linguistic noise. Although large language models (LLMs) improve semantic extraction, their computational cost limits routine deployment in resource-constrained public health settings.

**Objective:**

This study proposes and evaluates a hybrid framework that distills knowledge from an LLM (DeepSeek) into a lightweight bidirectional encoder representations from transformers (BERT)–based classifier for real-time influenza surveillance using Weibo data from Guangdong Province, China, during the 2025 influenza season. The study further assesses the temporal alignment between the derived signal and official influenza-like illness (ILI) surveillance data.

**Methods:**

A total of 389,959 Weibo posts from epidemiological weeks 7 to 50 (February 10 to December 14, 2025) were collected using a domain-specific keyword strategy. After multistage filtering, 99,158 posts were retained for analysis. A DeepSeek-Chat model was used to generate pseudo-labels for 2500 posts via zero-shot prompting, which were evaluated against expert annotations (Cohen κ=0.846) and used to fine-tune a Chinese-RoBERTa-WWM-ext classifier (a robustly optimized BERT pretraining approach). A chronological train-validation-test split was applied to reduce temporal leakage. The trained model was used to construct a weekly Weibo Flu Index. This index was compared with keyword-based and Term Frequency–Inverse Document Frequency (TF-IDF) + logistic regression baselines using time-lag Pearson correlation (0‐5 wk) against official ILI% data. Savitzky-Golay smoothing and second-derivative analysis were applied to identify epidemic inflection points and peak timing.

**Results:**

The classifier achieved an accuracy of 85.1% and an *F*_1_-score of 0.800 on a chronologically held-out test set, identifying 29,779 posts (30.0%) as ILI self-reports among 99,158 posts. The BERT-derived index showed the strongest correlation with official ILI% at lag 0 (*r*=0.523, 95% CI 0.268‐0.710; *R*²=0.274) and lag 1 (*r*=0.446, 95% CI 0.169‐0.658), compared with the keyword baseline (lag 0: *r*=0.481, *R*²=0.231) and TF-IDF baseline (lag 0: *r*=0.185, *R²*=0.034). Inflection analysis indicated that the Weibo-derived signal preceded both the epidemic rise (week 44 vs week 45) and peak (week 48 vs week 49) in official ILI data, corresponding to an approximate 1-week lead at both transition points.

**Conclusions:**

The results suggest that a DeepSeek-augmented lightweight BERT classifier can extract meaningful influenza-related signals from noisy Chinese social media data and produce a surveillance index that shows an approximate 1-week lead relative to official ILI reporting, with improved performance over keyword-based and TF-IDF approaches. This framework provides a proof-of-concept for cost-effective real-time influenza nowcasting using social media data.

## Introduction

Influenza surveillance remains a cornerstone of epidemic monitoring, but traditional sentinel-based systems commonly experience reporting delays of 1 to 2 weeks, limiting timely public health response and resource allocation [1]. Such delays can hinder timely public health response and weaken resource allocation during periods of rising transmission, as demonstrated by comparative evaluations of China’s national influenza surveillance systems showing persistent diagnostic and administrative lags across sentinel, notifiable disease, and virological reporting channels [2]. The need for faster surveillance has become more urgent in the context of the severe 2024 to 2025 influenza season and the postpandemic resurgence of influenza activity in Asia [3,4].

Social media platforms have emerged as promising complementary data sources because they capture health-related discourse in real time [5]. Prior studies suggest that social media signals may precede official surveillance reports; for instance, analysis of approximately 15 million Weibo posts demonstrated that semantically classified “sick posts” could predict COVID-19 case counts in mainland China up to 14 days ahead of official statistics [6]. However, the usefulness of these data is often undermined by substantial linguistic noise; analyses of Chinese-language Weibo posts during influenza seasons indicate that over 80% of retrieved posts contain nonpersonal commentary, media-driven discourse, or emotionally charged content unrelated to individual illness experience [7]. This limitation is especially problematic for keyword-based approaches, which often fail to capture the semantic complexity of health-related language [8]. The failure of Google Flu Trends remains a classic reminder that digital surveillance can become misleading when large-scale signals are interpreted without adequate semantic validation [9,10]. That failure was primarily driven by the conflation of media-amplified search behavior with genuine symptom incidence, a dynamic that is also plausible in social media contexts where breaking news or public anxiety can inflate platform activity independently of true infection burden. The present study directly addresses this limitation. Rather than relying on raw post volume or keyword frequency, we use a fine-tuned semantic classifier designed to distinguish first-person symptom expressions from media commentary, institutional announcements, and emotionally reactive posts. This distinction serves as a key methodological safeguard against media-amplification bias that has affected earlier digital surveillance efforts.

Recent advances in large language models (LLMs) have improved the extraction of meaningful public health information from noisy text [9]. However, direct deployment of large-scale models in operational surveillance systems remains constrained by substantial computational and financial costs, which pose challenges for public health agencies in resource-limited settings [10]. Knowledge distillation offers a practical alternative by transferring knowledge from a large teacher model to a smaller student model, enabling deployment-ready classifiers without continuous reliance on API-based inference [11]. In this study, DeepSeek functioned as the teacher model, generating binary labels indicating whether a post was influenza-related or non–influenza-related. These labels were used as high-confidence pseudo-labels for bidirectional encoder representations from transformers (BERT) fine-tuning. Among lightweight architectures, BERT-based models are widely used because they capture contextual semantics while remaining feasible for real-world deployment in settings where continuous access to large-scale model inference is not guaranteed [12].

In this study, we developed a real-time influenza surveillance framework using Weibo data from Guangdong Province during the 2025 influenza season. Given the absence of large-scale annotated Chinese health corpora and the high cost of deploying LLMs in public health settings, we used DeepSeek as a teacher model to generate high-quality training annotations for a lightweight BERT-based classifier, suitable for low-resource and operationally constrained surveillance settings. We then constructed a Weibo-based semantic influenza index and examined its temporal alignment with official influenza-like illness (ILI) data.

This study makes 4 contributions. First, it provides a proof-of-concept demonstration that semantic filtering via LLM-assisted distillation improves signal quality compared with keyword-based approaches in Chinese-language social media. Second, it characterizes the temporal co-variation between social media signals and official ILI surveillance data using cross-lagged correlation analysis across lags of 0 to 5 weeks. Third, it applies a curvature-based inflection detection method to independently verify the timing of epidemic escalation and peak signals, revealing a consistent 1-week lead of the Weibo-derived index over official ILI at both the rising and peak phases. Fourth, it proposes a practical framework for real-time digital surveillance in settings where labeled training data are scarce.

These findings should be interpreted as preliminary evidence and require validation across multiple influenza seasons prior to operational deployment.

## Methods

### Study Design and Data Acquisition

This retrospective study analyzed the 2025 influenza season in Guangdong Province, China, covering epidemiological weeks 7 to 50 from February 10 to December 14, 2025. We used Sina Weibo as the primary data source due to its high user penetration in the region. Data collection and processing adhered to Weibo’s Terms of Service and the Personal Information Protection Law of China.

A domain-specific keyword taxonomy was developed based on the *Diagnosis and Treatment Protocol for Influenza (2025 Edition*) and guidelines from the Guangdong Provincial Center for Disease Control and Prevention (CDC). The taxonomy used a hierarchical structure comprising core terminology (eg, “H1N1”), clinical symptoms (eg, “high fever”), and treatment behaviors (eg, “oseltamivir”).

### Data Preprocessing and Filtering

From an initial corpus of 389,959 posts, we applied a multistage filtering pipeline to identify verifiable, first-person reports of ILI. Posts were included if they met at least one of the following criteria: (1) self-reported acute respiratory or systemic symptoms (eg, fever ≥38 °C, cough, or sore throat) occurring within the preceding 48 hours, (2) explicit mention of a clinical diagnosis or a positive antigen test, or (3) descriptions of specific health care–seeking behaviors.

Posts were excluded if they were duplicates (n=67,234), institutional or bot-generated content (n=52,339), commercial advertisements, or news reports. After applying these exclusion criteria and manual annotation guidelines, the final analytic dataset comprised 99,158 posts (retention rate: 25.4%). The overall methodological workflow, spanning data preprocessing through model development, is illustrated in Figure 1.

**Figure 1.**
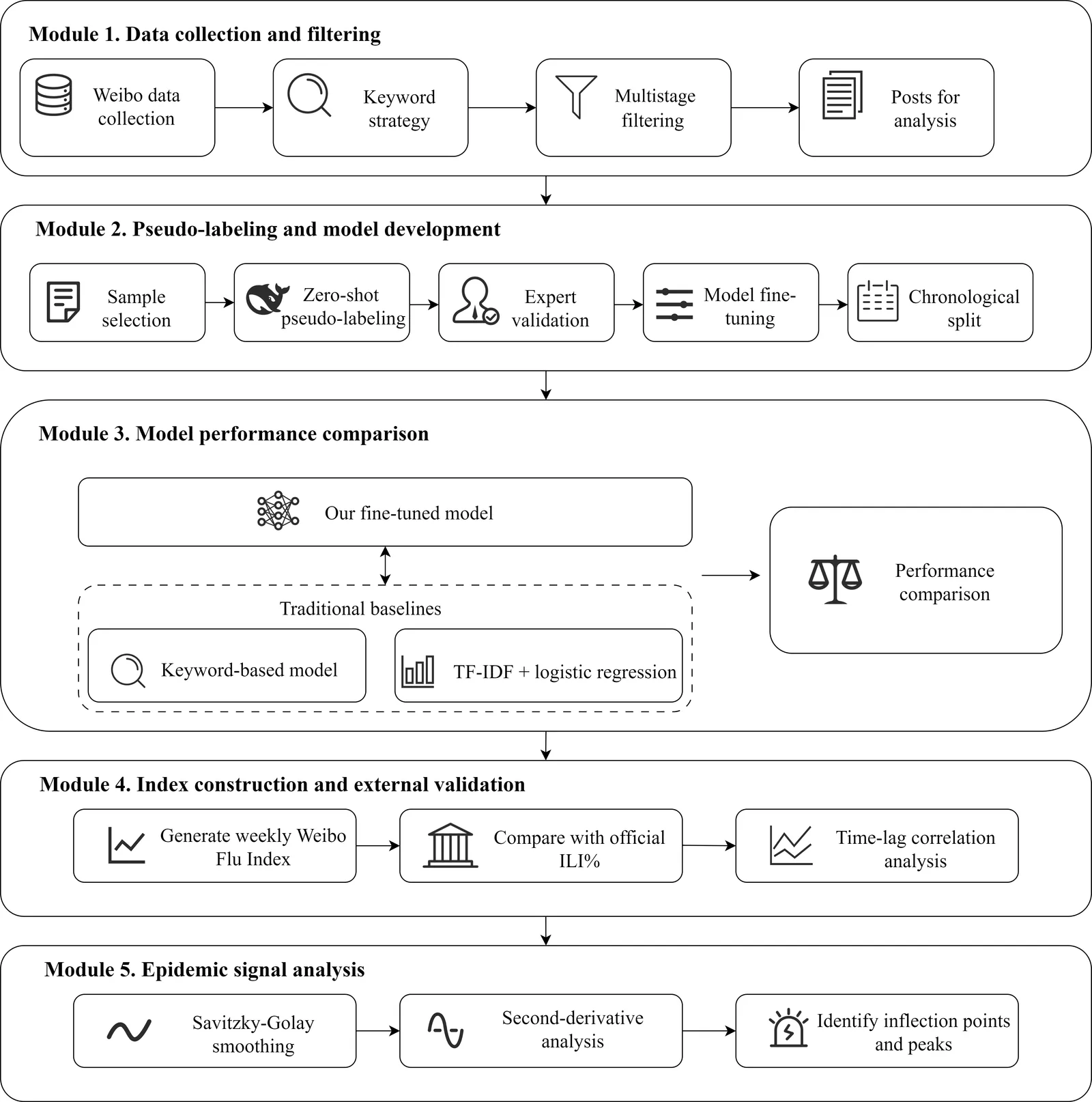
Study workflow for constructing and evaluating the Weibo Flu Index. ILI: Influenza-Like Illness; TF-IDF: Term Frequency–Inverse Document Frequency.

### The DeepSeek-Augmented Offline Knowledge Distillation Framework

To process the large volume of unstructured text efficiently, we implemented a “Teacher-Student” offline knowledge distillation framework using pseudo-labels generated by the teacher model.

#### Teacher Model Annotation (DeepSeek-Chat)

We used DeepSeek-Chat through the DeepSeek API (model identifier “deepseek-chat”) between December 2025 and early January 2026. According to the provider’s change log, the identifier pointed to DeepSeek-V3.2 in nonthinking mode during this period. It has since been redirected to other models and is scheduled for discontinuation, so the annotation cannot be rerun on the same end point.

We annotated a random sample of 2500 posts from the analytic corpus with a zero-shot prompt. Each post was submitted in its own request with no conversation history, using 1 system message that held the labeling rules and 1 user message that held the post text. The system message asked the model to act as an epidemiologist monitoring influenza A(H1N1) and A(H3N2) and to follow 3 steps: look for evidence of ILI, check for exclusions, and assign label 1 only if there was evidence and no exclusion; otherwise, label 0. Evidence came from 3 sets: a diagnosis, a positive antigen test, an antiviral drug (oseltamivir, baloxavir marboxil), or fever (set A); symptom patterns typical of H1N1, such as variable fever, early and prominent cough, marked sore throat, nasal symptoms, vomiting, abdominal pain or diarrhea, and early shortness of breath (set B); and patterns typical of H3N2, such as abrupt fever of 39‐40 °C or above, severe muscle and bone pain, splitting headache, severe chills, extreme fatigue, dry tearing cough, and a protracted course (set C). Five exclusions always led to label 0: an individual other than the author or an immediate family member; a time other than the current illness; rhetorical or nonpathological use of symptom words (eg, “so handsome it gives me a fever”); content limited to vaccines, stockpiling medicine, news, or worry; and a clearly different cause, such as gastroenteritis without respiratory symptoms, COVID-19, dysmenorrhea, an ordinary cold, or rhinitis. The model returned a JSON object with the label and a short reason. We gave no labeled examples and no instruction for step-by-step reasoning. Because a current, self-reported fever alone satisfied set A, positive posts denote self-reported ILI and not laboratory-confirmed influenza.

To check the reliability of the pseudo-labels, 2 annotators with a public health or clinical background labeled a random subsample of 200 of the 2500 posts, using the same definition as the prompt. They worked independently without seeing the DeepSeek labels, and then discussed their disagreements to reach a consensus label (58 positive, 142 negative). Cohen *κ* was 0.846; against the consensus, the DeepSeek labels had an accuracy of 91.5% (95% CI 87.5‐95.0), a precision of 0.815, a recall of 0.914, and an *F*_1_-score of 0.862 (CIs by percentile bootstrap). DeepSeek-Chat labeled 65 posts as positive, and 17 of its labels differed from the consensus (12 false positives and 5 false negatives). Because the prompt was adjusted on this same subsample, these figures are likely to be optimistic.

#### Student Model Training and Evaluation (BERT)

We use the Chinese-RoBERTa-WWM-ext (a robustly optimized BERT pretraining approach) as the lightweight student model, parameterized by θ. To prevent temporal data leakage, the 2500 annotated posts were split chronologically by posting date into training (n=1750, 70%; February 12-October 29, 2025), validation (n=375, 15%; October 29-November 23, 2025), and test (n=375, 15%; November 23-December 14, 2025) sets. The fine-tuned student model was evaluated on the chronologically held-out test set, yielding an accuracy of 85.1% and an *F*_1_-score of 0.800. We performed semantic subtype annotation for error analysis. To further characterize the differences between the keyword baseline and the BERT framework, all posts in the chronologically held-out test set (n=375) were manually reviewed and assigned to semantic subtypes for post hoc error analysis. The subtype scheme included true ILI symptom, general observation, sarcasm or joke, sentiment mismatch, health anxiety, advertisement or irrelevant content, and other unclassified posts. These subtype annotations were used only for descriptive post hoc error analysis (reported in the Results section) and were not used for model training or threshold selection.

#### Mathematical Formulation

The surveillance task is defined as a binary classification mapping a post *x_i_* to a label *y_i_* ∈ {0, 1}. The student model is trained to minimize the cross-entropy loss *L* relative to the teacher’s pseudo-labels $y_{i}^{T}$:


$$\theta^{\text{*}}\text{=}\underset{\theta }{argmin}\sum_{\left(x_{i},y_{i}^{T}\right)\in D_{train}}L\left(f\left(x_{i};\theta \right),y_{i}^{T}\right)$$

Training was executed using the Hugging Face Transformers library (Python 3.12) with the AdamW optimizer (learning rate 2e-5, batch size 32) for 3 epochs. Posts were truncated to 128 tokens, and we kept the epoch with the lowest validation loss (epoch 3). No threshold was tuned on any dataset. A summary of the key hyperparameters used for both the DeepSeek annotation process and BERT fine-tuning is provided in Table 1.

**Table 1.** Key hyperparameters for DeepSeek annotation and bidirectional encoder representations from transformers (BERT) fine-tuning.

Component and parameter	Value
DeepSeek-Chat annotation	
Prompting strategy	Zero-shot
Temperature	0
Annotated subset size	2500 posts
Human validation subsample	200 posts (Cohen κ=0.846)
Chinese-RoBERTa^a^-WWM-ext fine-tuning	
Optimizer	AdamW
Learning rate	2.00E-05
Batch size	32
Epochs	3
Classification threshold	0.5
Framework	Hugging Face Transformers (Python 3.12)

a RoBERTa: a robustly optimized BERT pretraining approach.

### Construction of the Weibo Flu Index

The fine-tuned BERT classifier was deployed to infer labels for the entire filtered corpus (n=99,158). A post was classified as a “genuine ILI self-report” if the predicted probability of the positive class exceeded a threshold of 0.5.

To quantify the intensity of social media discussions, we constructed the Weibo Flu Index, normalized to account for platform activity fluctuations:


$$I_{t}\text{=}\frac{N_{pos,t}}{N_{total,t}}\times 1000$$

where *I_t_* is the index for week *t*, *N*_pos_*_, t_* is the count of semantically validated positive posts, and *N*_total_*_, t_* is the total volume of collected posts for that week. To ensure methodological comparability, we constructed 2 baseline indices using the same filtered corpus (n=99,158 posts). First, a lexicon-based keyword baseline was constructed by including posts containing at least 1 first-person symptom keyword (eg, terms corresponding to fever, high fever, cough, sore throat, fatigue, headache, myalgia, runny nose, influenza A, and influenza B) and excluding posts containing media-reporting or third-person framing terms (eg, terms corresponding to as reported, experts stated, and data show). Second, a Term Frequency–Inverse Document Frequency (TF-IDF)+logistic regression baseline was constructed by training a TF-IDF vectorizer (max features=5000; unigrams and bigrams) and a logistic regression classifier (C=1.0; max iterations =1000; random seed=42) on the same 2500 chronologically split annotated posts. Weekly counts from both baselines were normalized using the same formula as the BERT-derived index.

### Statistical Validation and Nowcasting

Official validation data, defined as the weekly ILI percentage (ILI%), was obtained from the Guangdong Provincial CDC sentinel surveillance network. We assessed the temporal relationship between the Weibo Flu Index and official ILI% using time-lag cross-correlation analysis. Pearson correlation coefficients (*r*) were calculated for time lags of 0 to 5 weeks on first-differenced, stationary time series. CIs (95%) were estimated via Fisher *z*-transformation. This analysis was conducted for all 3 indices (BERT-derived, TF-IDF baseline, and keyword baseline) to enable direct methodological comparison. To identify the timing of rising inflection and peak signal points in both the Weibo-derived index and official ILI series, we applied Savitzky-Golay smoothing (window length=7, polynomial order=2) followed by first- and second-derivative analysis, defining the rising inflection as the week of maximum second-derivative during the escalation phase (weeks 43‐47) and the peak as the week of maximum signal value (weeks 44‐50).

### Ethical Considerations

This study was approved by the Institutional Review Board of the authors’ institution (approval number 2025‐102), with a waiver of informed consent due to its retrospective, noninterventional design using publicly available posts from Weibo. The study complies with Articles 13 and 27 of the Personal Information Protection Law of the People’s Republic of China, which permit the use of publicly available personal information without separate consent under conditions that protect individual rights. Data were collected via the Weibo Open Platform API in accordance with the platform’s Terms of Service. All direct identifiers (including usernames, user IDs, avatars, and IP-based geolocation, reduced to provincial level) were removed or irreversibly transformed using salted hashing, with mapping keys stored on a separate access-restricted server. Data were processed and stored on servers located in mainland China, and no data were transferred outside China. Only aggregated results are reported, and no individual-level posts are disclosed. The study posed minimal risk, and no reidentification was attempted. In accordance with JMIR data-sharing policies and applicable regulations, raw data are not publicly available due to legal and ethical constraints, but aggregated outputs and analysis code are available from the corresponding author upon reasonable request.

## Results

### Descriptive Statistical Analysis

From the initial data collection, 99,158 posts spanning epidemiological weeks 7 to 50 were retained for semantic analysis. The DeepSeek-augmented BERT classification revealed a substantial class imbalance within this keyword-filtered corpus: 29,779/99,158 (30.0%) posts were identified as genuine ILI self-reports (label 1), while 69,379/99,158 (70.0%) posts were classified as linguistic noise (label 0). This high proportion of noise (69,379/99,158, approximately 70%) highlights the limitations of simple keyword matching and supports the need for semantic filtering in social media surveillance.

Temporally, valid posts averaged 677 per week and increased markedly during the winter season, peaking at 2150 posts in week 48, 1 week prior to the official ILI peak (13.35%) observed in week 49.

### Performance of the DeepSeek-Augmented Classifier

The knowledge distillation strategy yielded a robust classifier. The fine-tuned Chinese-RoBERTa-WWM-ext model achieved an accuracy of 85.1% and an *F*_1_-score of 0.800 on a chronologically held-out test set (n=375) from November to December 2025. The area under the receiver operating characteristic curve was 0.921 (95% CI 0.892‐0.946), and the area under the precision-recall curve was 0.862 (95% CI 0.804‐0.912). Performance was stable across thresholds: between 0.30 and 0.70, accuracy against the DeepSeek labels ranged from 0.835 to 0.853, and the *F*_1_-score ranged from 0.763 to 0.810. This is expected because most posts received a predicted probability close to 0 or 1 (165 of 375 test posts below 0.05 and 33 above 0.95). These results suggest effective knowledge transfer from the teacher model to the student model.

The model’s discriminative ability is further illustrated by the t-SNE (t-distributed stochastic neighbor embedding) projection of the embedding space (Figure 2). The pretrained baseline exhibits substantial overlap between classes (Figure 2A), whereas the fine-tuned model demonstrates clearer separation between genuine ILI self-reports and linguistic noise (Figure 2B), indicating improved class separability after fine-tuning.

**Figure 2.**
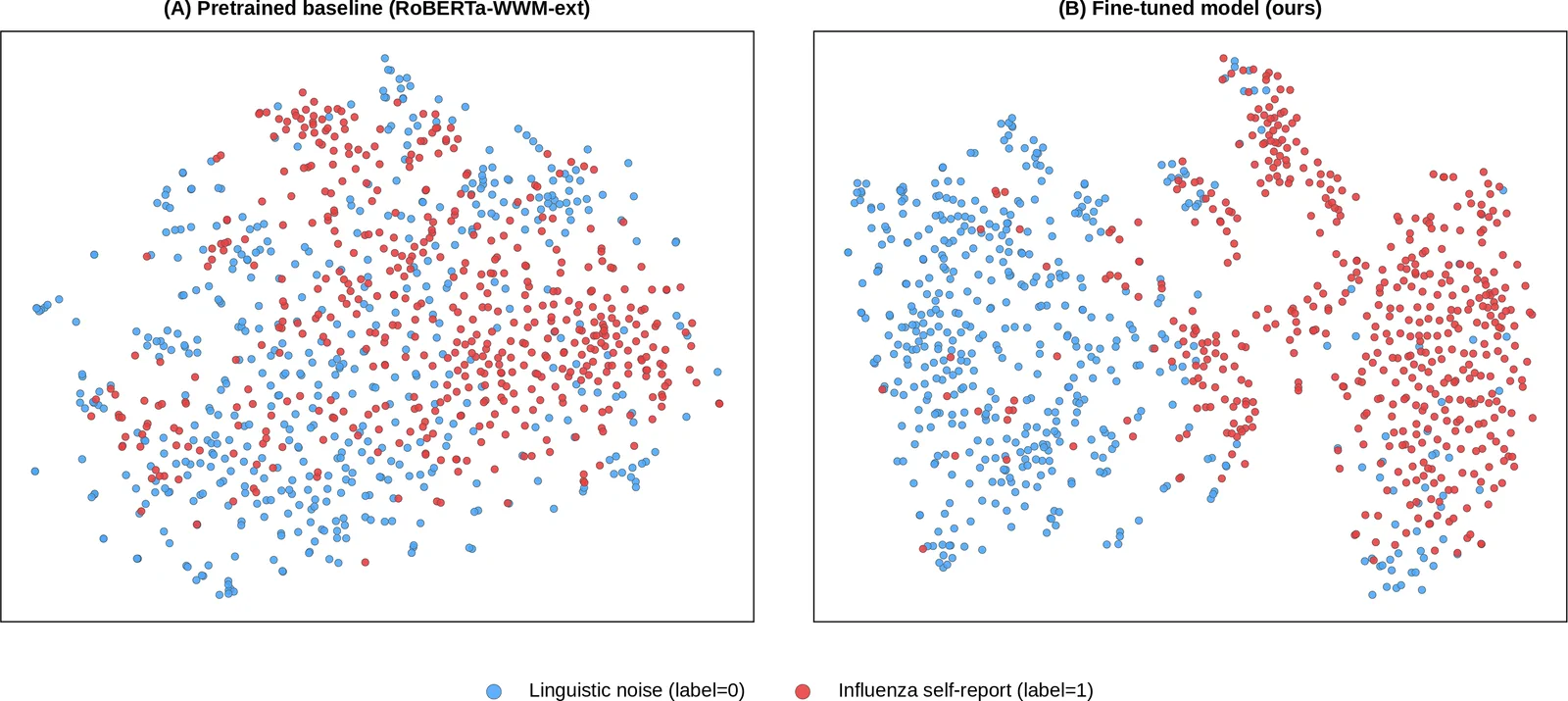
Comparative t-SNE (t-distributed stochastic neighbor embedding) visualization of semantic feature representations before and after fine-tuning. (A) Pretrained baseline (RoBERTa-WWM-ext): the embedding space of the pretrained model shows substantial overlap between genuine influenza self-reports (red) and linguistic noise (blue), indicating limited domain-specific discriminative capability. (B) Fine-tuned model (ours): after supervised fine-tuning, the model shows clearer separation between the 2 classes. RoBERTa: a robustly optimized BERT pretraining approach.

### Comparison With Keyword and TF-IDF Baselines

As shown in Figure 3, the DeepSeek-augmented BERT index showed the strongest association with official ILI% at lag 0 and lag 1, outperforming both baseline approaches at these shorter time horizons. At lag 0, the BERT index achieved the highest correlation with official ILI% (*r*=0.523), exceeding the keyword baseline (*r*=0.481) and the TF-IDF baseline (*r*=0.185). Correlation patterns varied across methods, with the BERT-derived index showing the strongest short-lag associations and weaker correlations at longer lags (Table 2).

**Figure 3.**
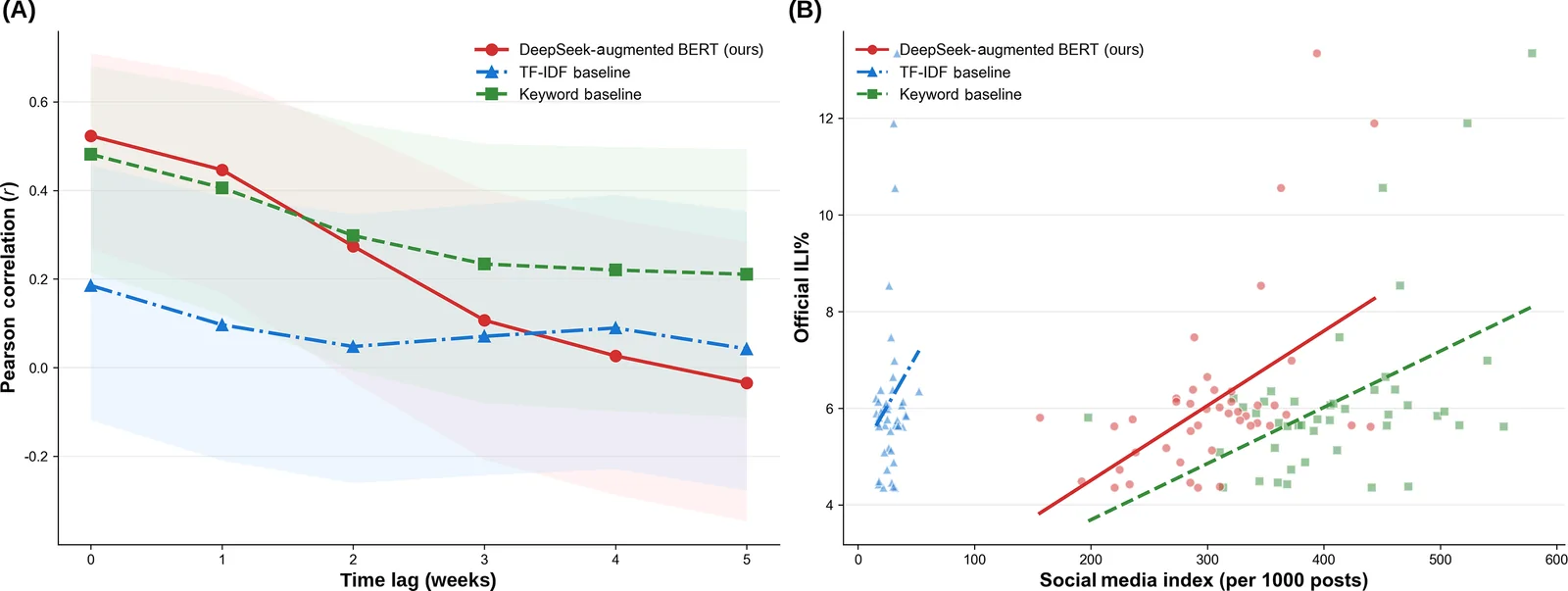
Comparative performance of the DeepSeek-augmented BERT index and baseline models across time lags. (A) Pearson correlation coefficients (*R*) between each social media–based index and official ILI% across time lags from 0 to +5 weeks, with 95% CIs denoted by shaded areas. (B) Scatter plots at lag 0 illustrating the relationship between each index and official ILI%. Note that the Term Frequency–Inverse Document Frequency (TF-IDF) baseline exhibits a substantially narrower range of index values compared to the bidirectional encoder representations from transformers (BERT) and keyword-based methods. ILI: influenza-like illness.

**Table 2.** Temporal correlation between social media indices and official influenza-like illness (ILI) across time lags.

Model	Lag 0, *r* (95% CI)	Lag 1, *r* (95% CI)	Lag 2, *r* (95% CI)	Lag 3, *r* (95% CI)	Lag 4, *r* (95% CI)	Lag 5, *r* (95% CI)
DeepSeek-augmented BERT^a^	0.523^a^ (0.268 to 0.710)	0.446^b^ (0.169 to 0.658)	0.274 (–0.033 to 0.534)	0.107 (–0.208 to 0.401)	0.027 (–0.287 to 0.335)	–0.035 (–0.346 to 0.284)
Keyword baseline	0.481^b^ (0.215 to 0.681)	0.406^b^ (0.120 to 0.630)	0.298 (–0.006 to 0.552)	0.234 (–0.080 to 0.505)	0.220 (–0.098 to 0.498)	0.211 (–0.112 to 0.493)
TF-IDF^c^ baseline	0.185 (–0.118 to 0.457)	0.097 (–0.210 to 0.386)	0.048 (–0.260 to 0.347)	0.071 (–0.242 to 0.370)	0.089 (–0.228 to 0.390)	0.042 (–0.277 to 0.353)

a BERT: bidirectional encoder representations from transformers.

b *P*<.01. All other correlations were not statistically significant at *P*<.05.

c TF-IDF: Term Frequency–Inverse Document Frequency.

Scatter plots in the right panel of Figure 3 further illustrate this distinction. At lag 0, the BERT-derived signal explained 27.4% of the variance in official ILI% (*R*²=0.274), compared with 23.1% for the keyword baseline and 3.4% for the TF-IDF baseline, indicating that the BERT-derived signal captured a larger share of the variance in official ILI% than the 2 baseline methods.

Unlike simple keyword matching, which applies uniform rules to influenza-related terms, the BERT framework demonstrated a broader semantic advantage over the keyword baseline (Table 3). Specifically, BERT not only reduced false positives arising from general observations and irrelevant content but also improved the identification of genuine ILI symptom posts that were missed by keyword matching. This finding highlights the importance of semantic filtering in social media epidemiology, where authentic illness self-reports are often intermixed with observational, affective, and off-topic discourse. On the chronologically held-out test set (n=375), the BERT framework achieved an overall accuracy of 85.1%, compared with 64.5% for the keyword baseline, corresponding to an absolute improvement of 20.5 percentage points (Table 3). The subtype-based analysis showed that this advantage was distributed across multiple semantic categories rather than being limited to a few illustrative examples. The largest gains were observed for advertisement or irrelevant posts, health anxiety posts, and other unclassified posts. Mechanistically, BERT reduced false positives arising from general observations and irrelevant content, while also recovering genuine ILI symptom posts that were missed by keyword matching.

**Table 3.** Semantic subtype-based error analysis comparing the keyword baseline and the bidirectional encoder representations from transformers (BERT) framework on the chronologically held-out test set (n=375).

Subtype	Posts, n	Keyword, n (%)	BERT, n (%)	A^a^, n (%)	B^b^, n (%)	Both wrong^c^, n (%)
True ILI^d^ symptom	125	82 (65.6)	104 (83.2)	0 (0)	37 (29.6)	6 (4.8)
General observation	124	76 (61.3)	101 (81.5)	34 (27.4)	0 (0)	14 (11.3)
Other unclassified	45	34 (75.6)	45 (100)	11 (24.4)	0 (0)	0 (0)
Ad/irrelevant	40	28 (70)	40 (100)	12 (30)	0 (0)	0 (0)
Sentiment mismatch	16	12 (75.0)	13 (81.2)	3 (18.8)	0 (0)	1 (6.2)
Sarcasm/joke	14	7 (50)	10 (71.4)	3 (21.4)	1 (7.1)	3 (21.4)
Health anxiety	11	3 (27.3)	6 (54.5)	3 (27.3)	0 (0)	5 (45.5)
Total	375	242 (64.5)	319 (85.1)	66 (17.6)	38 (10.1)	29 (7.7)

a Posts incorrectly classified as positive by the keyword baseline but correctly rejected by the bidirectional encoder representations from transformers framework.

b Posts missed by the keyword baseline but correctly identified as positive by the bidirectional encoder representations from transformers framework.

c Posts misclassified by both methods.

d ILI: influenza-like illness.

### Correlation Analysis Between Weibo Posts and Official ILI%

These quantitative findings are visually corroborated in Figure 4, which illustrates the temporal alignment between the BERT-filtered Weibo signal and official ILI data across epidemiological weeks 7 to 50. During the early study period (weeks 7‐26), the shifted Weibo signal remained below the official ILI trend, as reflected by predominantly negative deviations in the lower panel. From approximately week 27 onward, the standardized deviation became positive and remained elevated throughout the epidemic escalation phase, indicating that the Weibo signal began to track and intermittently exceed the official ILI trend. To formally identify the timing of key epidemic transitions, we applied Savitzky-Golay smoothing followed by first- and second-derivative analysis to both the Weibo signal and the official ILI series. The rising inflection point, defined as the week of maximum positive curvature (second derivative) during the escalation phase, was identified at week 44 for the Weibo signal and week 45 for official ILI, indicating that the Weibo-derived index detected the onset of rapid escalation 1 week ahead of the official surveillance data. Similarly, the peak signal volume occurred at week 48 for the Weibo index, compared with week 49 for the official ILI peak, confirming a consistent 1-week lead time at both the escalation onset and the epidemic peak. This temporal offset is consistent with the cross-correlation results in Table 2, where the strongest association between the Weibo index and official ILI was observed at lag 0 and lag 1.

**Figure 4.**
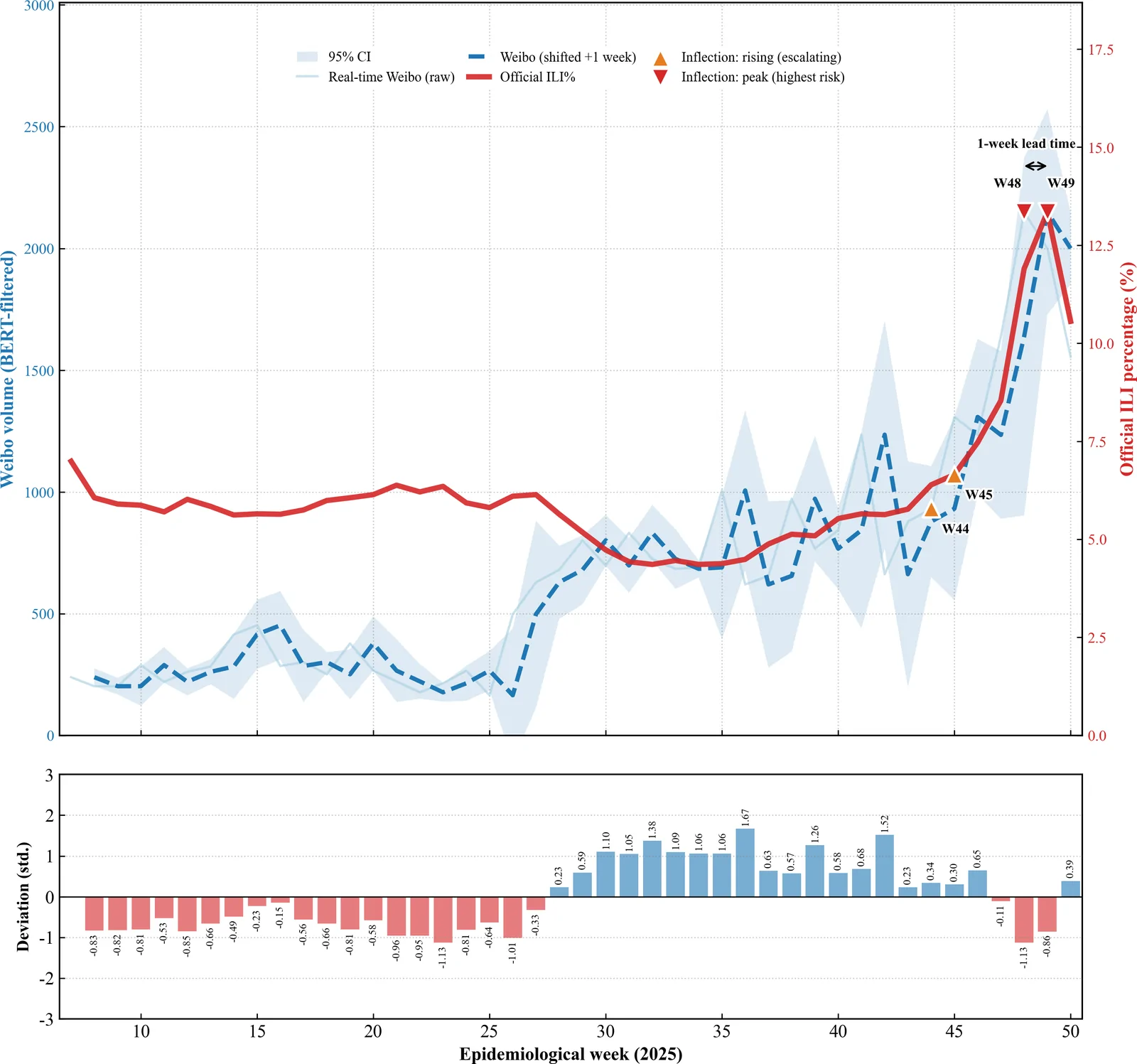
Temporal alignment between bidirectional encoder representations from transformers (BERT)–filtered Weibo influenza-related posts and official influenza-like illness (ILI) surveillance data, Guangdong Province, epidemiological weeks 7 to 50, 2025. Upper panel: real-time Weibo signal (light blue), 1-week shifted Weibo signal with 95% CI (dark blue dashed), and official ILI% (red). Triangles mark inflection points: rising escalation (week 44) and peak signal (week 48). Lower panel: standardized weekly deviation between the shifted Weibo signal and official ILI, with blue bars indicating weeks when the Weibo signal exceeded the ILI trend and red bars indicating the reverse.

## Discussion

### Main Research Findings

To address the temporal delay in traditional influenza surveillance, this study developed a hybrid nowcasting framework integrating LLMs models with a lightweight BERT classifier for signal extraction. Our analysis suggests that the BERT-derived Weibo Flu Index is strongly correlated with official ILI surveillance data and provides an approximate 1-week lead over standard reporting. Specifically, the inflection point indicating the onset of rapid escalation appeared in the Weibo signal at week 44, approximately 1 week earlier than the corresponding inflection in official ILI data at week 45; similarly, the peak occurred at week 48, preceding the official ILI peak at week 49. This consistent 1-week lead at both onset and peak further supports the potential of the Weibo-derived index as an early warning signal. Notably, the results suggest that semantic fidelity may be more informative than raw data volume; the keyword-based baseline, despite capturing more posts, showed lower predictive performance than the semantic model. By using DeepSeek for context-aware annotation, the framework helped reduce background linguistic noise and improve signal quality. These results suggest that the predictive use of social media may depend more on the quality of symptom identification than on the quantity of mentions, supporting its potential as a scalable framework for real-time disease monitoring.

### Comparison With Prior Work

Our finding that the Weibo-derived index may detect epidemic transitions ahead of official surveillance reporting is broadly consistent with prior digital epidemiology studies showing that online behavioral signals can complement traditional infectious disease monitoring systems [13]. For example, studies integrating search trends and social media data using seasonal autoregressive integrated moving average–based models have reported strong correlations with official case data across diverse settings, and improved performance compared with case-only baseline models [14]. These findings support the broader premise that digital signals, when appropriately validated, may serve as timely proxies for evolving epidemic activity.

A key contribution of this study is the observed advantage of semantic filtering over raw volume-based approaches. Although the keyword-based baseline captured a larger number of posts, it showed lower correlation with official ILI data than the DeepSeek-augmented BERT classifier, suggesting that post volume alone may not fully reflect underlying disease activity. This is consistent with prior work suggesting that context-aware transformer models such as BERT, RoBERTa, and BERTweet generally outperform lexicon-based or shallow text-mining approaches in health-related social media classification tasks, particularly in informal language settings [15,16]. Comparative studies of pretrained transformer architectures for social media health text classification further indicate that model design and domain adaptation are more influential than raw data volume in determining classification performance [16]. Our results extend this line of work, suggesting that refining these signals may improve accuracy. Beyond correlation-based evaluation, this study extends prior work by examining epidemic transitions using curvature-based inflection detection. Using Savitzky-Golay smoothing and second-derivative analysis, we observed that the Weibo-derived signal reached both its inflection point and peak approximately 1 week earlier than official ILI data. This is consistent with early warning signal research in infectious disease epidemiology, which emphasizes the potential of detecting critical transitions in surveillance time series before they are captured by conventional reporting systems [17]. Similarly, recent studies applying early warning frameworks to COVID-19 case data have shown that time-series–derived indicators can anticipate epidemic peaks ahead of official confirmation, supporting the feasibility of transition-based nowcasting approaches [18]. These findings provide additional support for transition-based time-series approaches for early epidemic detection in digital surveillance systems. The subtype-based error analysis further clarifies the mechanism of this advantage, showing that the BERT framework improved performance through both false-positive reduction in observational or irrelevant posts and improved recovery of genuine symptom self-reports missed by keyword matching.

This study also contributes to ongoing discussions on model complexity versus deployment feasibility in digital surveillance. While LLMs provide strong contextual understanding of health-related text, their computational and financial costs limit direct deployment in routine public health settings [10]. Knowledge distillation approaches, in which a large teacher model generates pseudo-labels for training a smaller student model, have been shown to reduce inference costs while maintaining comparable performance in text classification tasks [19]. Our results suggest the applicability of this approach to infectious disease surveillance, showing that a distilled lightweight BERT classifier trained on LLM-generated annotations can achieve strong agreement with official epidemiological data while remaining suitable for real-time, resource-constrained deployment [11,19]. This finding aligns with emerging evidence that AI-enabled surveillance systems leveraging open social media data may provide early epidemic signals ahead of formal outbreak confirmation in settings with limited or delayed reporting capacity [20]. These results suggest that combining LLMs with lightweight classifiers may offer a practical balance between predictive performance and deployment feasibility in real-world surveillance settings.

### Theoretical Implications

The performance gap observed between our semantic model and the keyword baseline highlights a central challenge in digital epidemiology, where linguistic noise (ie, posts discussing influenza-related information rather than personal illness) may dilute predictive signal [15]. Recent studies on transformer-based models for social media health monitoring suggest that context-aware text classification may help separate epidemiological signals from background linguistic noise and media-related chatter, particularly when models are trained on high-quality labeled data [15,16]. However, increasing data volume alone does not necessarily improve disease prediction accuracy [21]. Consistent with findings from Google Flu Trends, this study suggests that unfiltered, high-volume keyword data may be associated with reduced surveillance accuracy [22]. The lower performance of the keyword baseline further indicates a potential validity limitation, as search terms and social media mentions may reflect health care–seeking behavior and public awareness rather than true infection incidence, a pattern supported by quantitative evidence showing that search-volume peaks may lag behind community symptom onset by days to weeks [23]. Curvature-based inflection analysis showed that the semantically filtered Weibo signal preceded both the escalation onset and peak of official ILI activity by approximately 1 week. This finding is consistent with early warning signal literature suggesting that reducing linguistic noise may improve the temporal resolution of epidemic transition detection [17,18]. These results suggest that semantic context may play an important role in improving the interpretability of social media–based epidemiological signals compared with keyword-based approaches, indicating that the lack of semantic information may be a limitation in traditional keyword-driven methods.

### Practical Implications

From a public health perspective, influenza surveillance systems are affected by substantial reporting delays. Comparative analyses of China’s sentinel ILI system, the national notifiable disease reporting system, and virological surveillance during the 2021 to 2024 influenza seasons indicate that all 3 data sources exhibit delays that may limit their use for real-time detection of epidemic onset [2]. To address this limitation, this study proposes a scalable framework for real-time influenza nowcasting (this study). By integrating LLMs with lightweight BERT-based classifiers for deployment, the approach may improve contextual interpretation of social media data (eg, distinguishing “I have fever” from “Fever is a flu symptom”) (this study). Compared with keyword-based methods and computationally intensive LLM-only approaches [24], this framework may offer a balance between predictive performance and deployment feasibility, particularly in resource-constrained settings. In practice, the Weibo Flu Index could be integrated as a complementary early-warning signal within existing sentinel surveillance infrastructure, such as Guangdong CDC’s sentinel network, generated on the same weekly reporting cycle to allow direct cross-referencing with sentinel hospital data. Rather than replacing laboratory-confirmed sentinel reporting, the framework could function as a triggering mechanism: when the index exceeds a predefined threshold relative to historical baselines, it could prompt CDC staff to intensify case verification or issue preliminary alerts ahead of formal sentinel confirmation, thereby helping to narrow the 1 to 2 week reporting lag inherent in traditional surveillance channels.

### Limitations and Future Research

This study covers 1 province (Guangdong), 1 influenza season (2025), and 1 platform (Sina Weibo), so it remains uncertain whether the findings would hold in other settings. The weekly analysis rests on 44 observations from a single season, that is, 1 epidemic curve. The lagged correlations, the comparison with the baseline indices, and the timing of the rise and peak therefore describe how the index behaved in 1 wave. They do not show whether it would track surveillance data in other seasons, in seasons dominated by other subtypes or by influenza B, or in periods of low activity. The chronological hold-out also lies within the same season, and entirely within the autumn-winter surge (23 November to 14 December), when the share of positive posts (139/375, 37.1%) was higher than in the training period (434/1750, 24.8%). Test-set accuracy therefore mainly reflects performance at peak activity and does not directly tell us how well the classifier would transfer to other seasons, provinces, or platforms. Weibo users are also likely to differ from the general population in age, urbanicity, and health care use, which may limit how well the index represents population-level influenza incidence, and influenza seasonality, circulating strains, health-seeking behavior, and Weibo use vary among Chinese provinces, so the index may represent a different part of the population, or behave differently, elsewhere. In addition, the corpus is defined by a fixed set of influenza-related search terms, language use, and platform behavior change over time, so the classifier may need retraining. The teacher model end point is no longer available, so the labeling cannot be repeated exactly, and agreement with human labels was estimated on a subsample on which the prompt had been adjusted. The semantic subtype analysis in Table 3 was based on post hoc manual categorization of the held-out test set, and some categories contained relatively small numbers of posts, so these subtype-level findings should be interpreted as descriptive and may be sensitive to category definitions. The absence of meteorological and mobility variables may further limit the ability to capture broader transmission dynamics, and cross-correlation assesses temporal association only, so it may remain vulnerable to unmeasured confounding and shared external drivers and should not be interpreted as evidence of true causation. Generalizability needs to be tested on independently annotated data from other seasons, provinces, and platforms, and prospectively, with alert criteria fixed in advance. This study is a single-season feasibility evaluation, and further validation is needed before the index is used operationally.

Another limitation is that the framework does not differentiate influenza subtypes (eg, A[H1N1] vs A[H3N2]) because subtype-level laboratory confirmation was not available in the Weibo-derived data. Future studies incorporating sentinel laboratory subtyping data may help determine whether model performance varies across circulating strains.

The model also relied on hard pseudo-labels for student training rather than soft-label distillation, which may have resulted in the loss of uncertainty information from the teacher model. Future work could explore soft-label or confidence-weighted distillation to better preserve teacher uncertainty during knowledge transfer.

Future research should extend this framework using multiplatform and multiregion data, incorporate meteorological and mobility variables, and conduct multiseason validation to improve robustness and generalizability.

### Conclusions

The proposed framework detected epidemic transitions approximately 1 week ahead of official surveillance data, suggesting potential use for more timely resource allocation decisions and vaccination planning. The use of a distilled BERT classifier demonstrates that LLM-informed surveillance approaches may be implemented in a cost-effective manner, including in resource-constrained settings such as local CDC. This study provides a preliminary proof-of-concept for integrating LLMs with lightweight classifiers in digital disease surveillance. Future work should explore the integration of multisource data streams to further evaluate and enhance the robustness of early warning systems in public health surveillance.

## Abbreviations

BERT: bidirectional encoder representations from transformers
CDC: Center for Disease Control and Prevention
ILI: influenza-like illness
LLM: large language model
RoBERTa: a robustly optimized BERT pretraining approach
t-SNE: t-distributed stochastic neighbor embedding
TF-IDF: Term Frequency–Inverse Document Frequency
